# Peterson’s Ladies’ National Magazine

**Published:** 1863-10

**Authors:** 


*Peterson's Ladies' National Magazine.*—Whoever would be themselves, and have their children in the fashion, should take Peterson's Magazine; it is a very fashionable book, and always contains illustrations of the latest fashions. It also has fashionable literature from the first American authors, and always makes itself an attractive and welcome visitor.

